# Meetings of Societies

**Published:** 1938

**Authors:** 


### Meetings of Societies

---

The fourth meeting of the Session was held on Wednesday, 12th January, at 8.30 p.m., in the Physiological Lecture Theatre of the University.

Mr. C. A. Moore was in the chair, and seventy-eight members present.

Mr. A. Wilfrid Adams showed a case of lower left lobectomy for bronchiectasis.

Mr. A. L. d'Abreu gave a lecture entitled "**The Scope of Modern Surgery in Thoracic Disease,**" which was much appreciated.

---

The fifth meeting of the Session was held on Wednesday, 9th February, at 8.30 p.m. in the Physiological Lecture Theatre of the University.

Mr. C. A. Moore was in the chair and sixty-nine members were present.

Dr. J. Innes Noble read a paper on "**A Vaccine Treatment for Rheumatoid Arthritis,**" and Dr. G. L. Foss read a paper on "**Some Clinical Applications of the Male and Female Sex Hormones.**"

---

The sixth meeting of the Session was held on Wednesday, 9th March, at 8.30 p.m. in the Physiological Lecture Theatre of the University.

Mr. C. A. Moore was in the chair and fifty-two members were present.

Dr. H. J. Orr-Ewing read a paper entitled "**Problems of the School Doctor.**"

